# Intestine and spleen microbiota composition in healthy and diseased tilapia

**DOI:** 10.1186/s42523-022-00201-z

**Published:** 2022-08-13

**Authors:** Tamir Ofek, Maya Lalzar, Ido Izhaki, Malka Halpern

**Affiliations:** 1 Department of Evolutionary and Environmental Biology, Faculty of Natural Sciences, University of Haifa, 3498838 Haifa, Israel; 2 Central Fish Health Laboratory, Fishery and Aquaculture Department, Ministry of Agriculture and Rural Development, 1080300 Nir David, Israel; 3 Bioinformatics Service Unit, University of Haifa, 3498838 Haifa, Israel; 4 Department of Biology and Environment, Faculty of Natural Sciences, University of Haifa, 3600600 Oranim, Tivon, Israel

**Keywords:** Hybrid tilapia, Intestine and spleen microbiota composition, Intensive freshwater aquaculture, Pathogenic bacteria, Bacterial co-infection

## Abstract

Symbiotic bacteria within the gut microbiome of various organisms, including fish, provide the host with several functions that improve the immune system. Although the spleen plays an important role in the modulation of immune responses, the role of spleen microbiota in shaping the immune system is unclear. Our study aimed at understanding the relationship between fish health and microbiota composition in the intestine and spleen. Our model organism was the hybrid tilapia (*Oreochromis aureus* × *Oreochromis niloticus*). We sampled intestine and spleen from healthy and diseased adult tilapia and determined their microbiota composition by sequencing the 16S rRNA gene. Significant differences were found between the intestine and the spleen microbiota composition of healthy compared to diseased fish as well as between intestines and spleens of fish with the same health condition. The microbiota diversity of healthy fish compared to diseased fish was significantly different as well. In the intestine of healthy fish, *Cetobacterium* was the most abundant genus while *Mycoplasma* was the most abundant genus in the spleen. *Vibrio* was the most abundant genus in the intestine and spleen of diseased fish. Moreover, it seems that there is a co-infection interaction between *Vibrio* and *Aeromonas*, which was reflected in the spleen of diseased fish. While *Vibrio, Aeromonas* and *Streptococcus* were the probable pathogens in the diseased fish, the role of *Mycoplasma* as a pathogen of cultured hybrid tilapia remains uncertain. We conclude that the intestine and spleen microbiota composition is strongly related to the health condition of the fish.

**Supplementary Information:**

The online version contains supplementary material available at 10.1186/s42523-022-00201-z.

## Background

Fish gut microbiota plays a role in many aspects of fish physiology, which include feeding, digestion, metabolism, energy homeostasis, reproduction, and immune responses [1]. Fish gut microbiota composition has been well studied and includes many bacterial species. Among them are beneficial bacteria and pathogenic bacteria, which the gut of a variety fish species harbors [2]. Fish gut commensal microbiota can function as a barrier against pathogens by preventing colonization of external pathogens. Recent studies reported that *Cetobacterium* was highly abundant in healthy tilapia gut microbiota composition [3–5]. Nevertheless, some pathogens have developed strategies to overcome this barrier [6].

In fish, bone marrow and lymph nodes are not present, and the major immune organs are the thymus, the head kidney (bone marrow equivalent), and the spleen [7]. The spleen, which is present in almost all gnathostomes (jawed vertebrates), is regarded as a primordial secondary lymphoid organ in which the adaptive immune responses are generated [8]. Fish spleen filter peripheral blood and the filtration occurs through sheets of leucocytes. Furthermore, the fish spleen harbour melano-macrophages, which are arranged in clusters or dispersed and serve as dumping sites for all kinds of material [9]. Some isolations of pathogenic fish bacteria from tilapia spleens, in the past few years, were reported by Soto et al. [10] who isolated *Francisella* and Amal et al. [11] and El Latif et al. [12], who isolated *Aeromonas*. In these studies, molecular tools were used for bacterial identification.

Tilapia culture is one of the most developing and profitable trades in aquaculture. Tilapia are cultured in many countries due to their fast growth, large size and high protein content [13]. The total farmed tilapia harvest in 2020 was around 6.9 million tons [14]. Commercial production of hybrid tilapia includes several hybrids; the two of the most popular hybrids are the *Oreochromis aureus* × *Oreochromis mossambicus* hybrid and the *O. aureus* × *Oreochromis niloticus* hybrid [15]. The main tilapia hybrid in the Israeli aquaculture is the hybrid of *O. aureus,* which is endemic to Israel, and *O. niloticus.* This hybrid was created to produce all-male hybrids by interspecific hybridization; however, due to problems with the purity of the *O. niloticus* species, the progeny of the hybridization were only "nearly" all-male [16].

The increase in demand for aquaculture products has changed fish farms from extensive to semi-intensive or intensive fish farming. This raises fish stress and susceptibility to bacterial infections that cause mortalities and high economic losses [17]. Commensal bacteria that inhabit the fish microbiome do not usually infect their healthy hosts. However, under stress, they may relatively proliferate and cause disease [18]. Bacterial infections could also originate from infection by a primary pathogen that causes dysbiosis and allows opportunistic pathogens to infect the host [7].

To the best of our knowledge, there are only a few gut microbiota studies that compared healthy and diseased fish (Bozzi et al. [19], Ma et al. [20], Li et al. [21, 22] and [23]). Here we aimed to understand the relationship between tilapia microbiota (intestine and spleen) and fish pathology. Specifically, we addressed the question: Is there a difference between the microbiota of diseased and healthy hybrid tilapia? To answer this question, we studied the intestines and spleens of healthy and diseased hybrid tilapia (*O. aureus* × *O. niloticus*) that were sampled from intensive freshwater aquacultures in Spring Valley, Israel. Our results showed that there are significant differences between the intestine and the spleen microbiota composition of healthy hybrid tilapia compared to diseased hybrid tilapia. The results of this study provide characterization of the intestine and spleen microbiota of healthy and diseased hybrid tilapia in freshwater aquaculture. These data may help in developing molecular tools for assessing fish health and thus controlling fish bacterial diseases.

## Methods

### Fish samples

Fish were sampled from fishponds located in the Spring Valley (formerly Beit She'an Valley) in northeast Israel (Additional file 1: Fig. S1) that were stocked with hybrid tilapia, among other edible fish species. Most ponds are 1–3 acres, and each pond contains around 15,000 fish, on average, per acre. Fish that were sampled were brought to the Central Fish Health Laboratory for health examination (Fishery Department, Ministry of Agriculture and Rural Development, Kibbutz Nir David, Israel). Sampling took place between January 2018 and December 2018 and covered three seasons in the Spring Valley (winter: Dec–Feb, spring: Mar–Apr, summer: May–Oct). Intestine and spleen samples were taken from healthy hybrid tilapia fish (n = 14 fish) (11 intestine and 13 spleen samples), and from diseased hybrid tilapia fish (n = 22 fish) (22 intestine and 21 spleen samples), that showed external signs of disease. The external signs observed in the subset of diseased fish were as follows: 19 of the diseased fish showed skin hemorrhagic septicemia together with skin lesions and skin necrosis, while three of the diseased fish showed eye exophthalmos. Beside visual inspection, health status of all fish was assessed by parasitological and microbiological analyzes (more details can be found in Additional file 1: Table S1). We sampled only adult fish (> 100 g) that grow in large fishponds and/or reservoirs during the fattening stage (Additional file 1: Table S2). To confirm that the difference in the weight between diseased and healthy fish did not influence the microbiota composition, fish were divided into two groups; medium (100–500 g) and large (500–1100 g) size, and statistical analysis of alpha and beta diversity were calculated for the microbiota composition of the different size groups, for each organ.

### Fish feed

The tilapia feed on a pellet that was manufactured by two main blend institutes, Zemach Extrufeed Aqua (https://zemach-extrufeed.co.il) and Raanan Fish Feed (https://raanan-fishfeed.com). The tilapia pellet is made from poultry by-products, cereals and cereal by-products, seed oils and their by-products and fish oil. The pellet nutritional values are 30–35% crude protein, 4–6% crude fat, 5–5.5% crude fiber, 7–9% ash, 9.5–10% moisture, 1.2–1.7% calcium and 1–1.2% phosphorus.

### Intestine and spleen sampling

Samples of the intestine and spleen from healthy and diseased fish were taken separately in aseptic conditions with surgical instruments that were soaked in Ethanol (70%) and burned in flame. The samples were transferred into 2 ml sterile test tubes (three tubes for each sample) containing 750 µl of absolute ethanol and then kept, at −20 °C until DNA extraction.

### DNA extraction

To obtain DNA without ethanol residues, the tubes with the intestine and spleen samples were centrifuged for 30 min at room temperature, 12,000 rpm, and the ethanol was removed from the tubes. DNA was extracted from the samples as described previously by Laviad-Shitrit et al. [24], using a DNA isolation kit (DNeasy Blood and Tissue, Qiagen, Germany) according to the manufacturer’s instructions with minor modifications. To ensure DNA quality, the DNA quantity and quality were evaluated by NanoDrop (Thermo Scientific 1000). The extracted DNA samples were stored at −20 °C.

### Generation of the 16S rRNA amplicon library

A set of primers was used to amplify the V4 variable region of the 16S rRNA gene: CS1_515F (ACACTGACGACATGGTTCTACAGTGCCAGCMGCCGCGGTAA) CS2_806R (TACGGTAGCAGAGACTTGGTCTGGACTACHVGGGTWTCTAAT) (Sigma Aldrich, Israel) [25]. PCR amplification was performed using the EmeraldAmp MAX HS PCR Master Mix (Takara Bio Inc, Otsu, Shiga, Japan). The primers contained 5’ common sequence tags (known also as common sequence 1 and 2, CS1 and CS2). Amplicons were created using two-stage “targeted amplicon sequencing (TAS)” as described previously by Naqib et al. [26]. PCR was performed as described by Sela et al. [27]. Sterile DNA-free water was used as a negative control for DNA extraction and PCR amplification to verify that there was no contamination. No contamination was found.

### Illumina MiniSeq sequencing

Subsequently, a second PCR amplification was performed in 10 µl reactions in 96-well plates. A master-mix for the entire plate was made using MyTaq HS 2X master-mix (Bioline, London, UK). Each well received a separate primer pair with a unique 10-base barcode, obtained from the Access Array Barcode Library for Illumina (Fluidigm, South San Francisco, CA; Item# 100-4876). These AccessArray primers contained the CS1 and CS2 linkers at the 3’ ends of the oligonucleotides. The conditions for the second PCR and the procedure of the Illumina sequencing were performed as was described in Sela et al. [27].

Pooled, diluted libraries were sequenced on an Illumina MiniSeq instrument and analysed with Casava pipeline 1.8 (Illumina, San Diego, CA, USA). The reads were 150 nucleotides in length and PhiX DNA served as a spike-in control. Barcode sequences from Fluidigm were provided to the MiniSeq server, and sequences were automatically binned according to their 10-base multiplex identifier sequences. Raw reads were recovered as FASTQ files. The second PCR, library preparation, pooling, and sequencing were performed at the University of Illinois at Chicago Sequencing Core (UICSQC) within the Research Resources Center (RRC).

### Sequence analysis

In total, 268 files in fastq format were generated, corresponding to 67 samples, two pair-ends sequences for each sample (four files for each sample). All the samples were of high quality in both directions of sequencing. Sequence data were analyzed using the DADA2 pipeline [28]. A detailed description of the data processing is described in Laviad-Shitrit et al. [29]. Following the data processing, both runs were merged by sample, and amplicon sequence variants (ASVs) of non-bacterial origin (Archaea, chloroplast, mitochondria, and unclassified phyla), were filtered out.

Raw sequence data were submitted to the National Center for Biotechnology Information Sequence Read Archive (https://www.ncbi.nlm.nih.gov/bioproject/) under the BioProject accession number PRJNA753117.

### Statistical analysis

All statistical analyses were performed in R version 3.6.2 [30], unless otherwise specified. Data was subsampled to 8,000 sequences per sample and normalized before statistical analyses were performed. The rarefaction curve and beta diversity NMDS (Bray–Curtis index) PERMANOVA test, were calculated using MicrobiomeAnalyst [31]. Alpha diversity was calculated with the Shannon coefficient and compared between the intestine and the spleen and between healthy and diseased fish with an ART ANOVA test. Effects of fish health, organ type and the interaction between them on beta diversity were calculated using a PERMANOVA test. ASV linear discriminant analysis (LDA) scores were calculated by linear discriminant analysis effect size (LEfSe) in order to find the main ASVs that contributed to the variation between the two organs and between the two health conditions.

## Results

The 67 hybrid tilapia samples of healthy and diseased fish from fish ponds in Spring Valley, Israel, yielded, in total, 3,347,134 reads that were generated with an average of 49,957 (± 25,010) reads per sample and, an overall number of 5555 ASVs (Additional file 2: Data S1). Rarefaction curves of each sample reached an asymptotic level, suggesting that our sampling efforts were sufficient to obtain a full estimate of ASV richness (Additional file 1: Fig. S2).

### Beta diversity

**Fig. 1:**
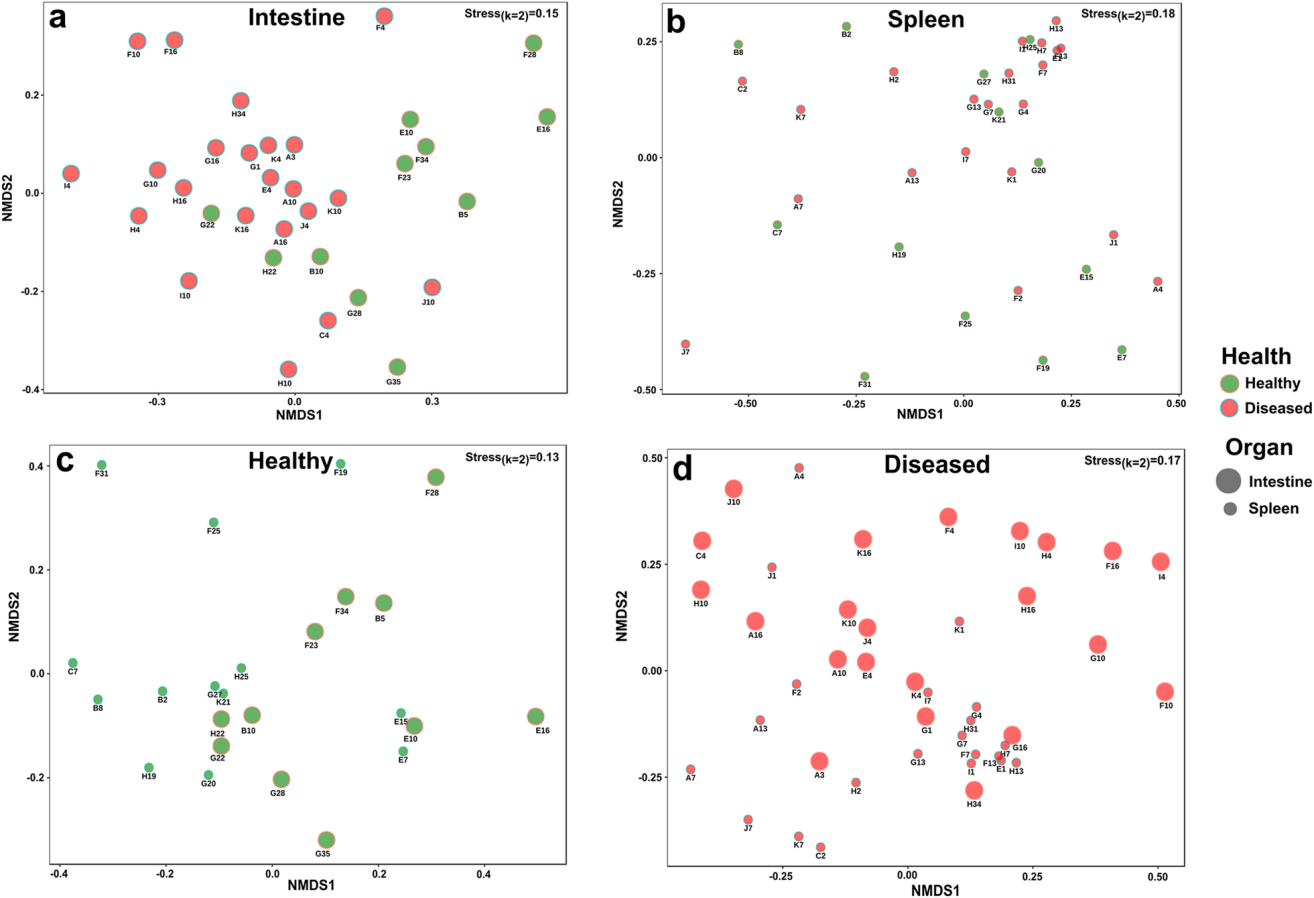
An NMDS plot (Bray–Curtis dissimilarity) of the microbiota composition of the samples from fish intestines (**a**) and spleens (**b**), from healthy and diseased fish. A comparison between intestines and spleens in the fish from the same health condition is presented in (**c**) (healthy fish) and (**d**) (diseased fish). More details regarding the fish sample identity can be found in Additional file 1: Table S3

The differences between the microbiota composition of the different organs and for the two health conditions were examined using the nonmetric multidimensional scaling (NMDS) (Fig. 1).

The results demonstrated that the bacterial community composition of the intestine of healthy fish significantly clustered separately from that of the diseased fish (Fig. 1a, Table 1). The same phenomenon was observed for the bacterial community of the spleen (Fig. 1b, Table 1). Moreover, the microbiota composition of the spleen and the intestine samples significantly clustered into two distinct groups, both in the healthy as well as in the diseased fish (Fig. 1c and d, Table 1). No significant differences in the microbiota composition were found between medium and large size fish, both for the spleen and intestine samples (*p* > 0.05) (Additional file 1: Table S2).

**Table 1:** Comparison between the microbiota composition of the different examined fish organs across health conditions

Sample type	Factor	F	R^2^	*p*
Intestine	Health*	4.40	0.12	< 0.001
Spleen	Health*	2.88	0.08	< 0.01
Healthy fish	Organ**	2.74	0.11	< 0.01
Diseased fish	Organ**	2.29	0.05	< 0.05

PERMANOVA tests demonstrated that there were significant differences between the microbiota compositions of the two organs in the same fish health condition and between the same organ in the healthy and the diseased fish (See also Fig. 1)

*Healthy versus diseased. **Intestine versus spleen

### Alpha diversity

**Fig. 2:**
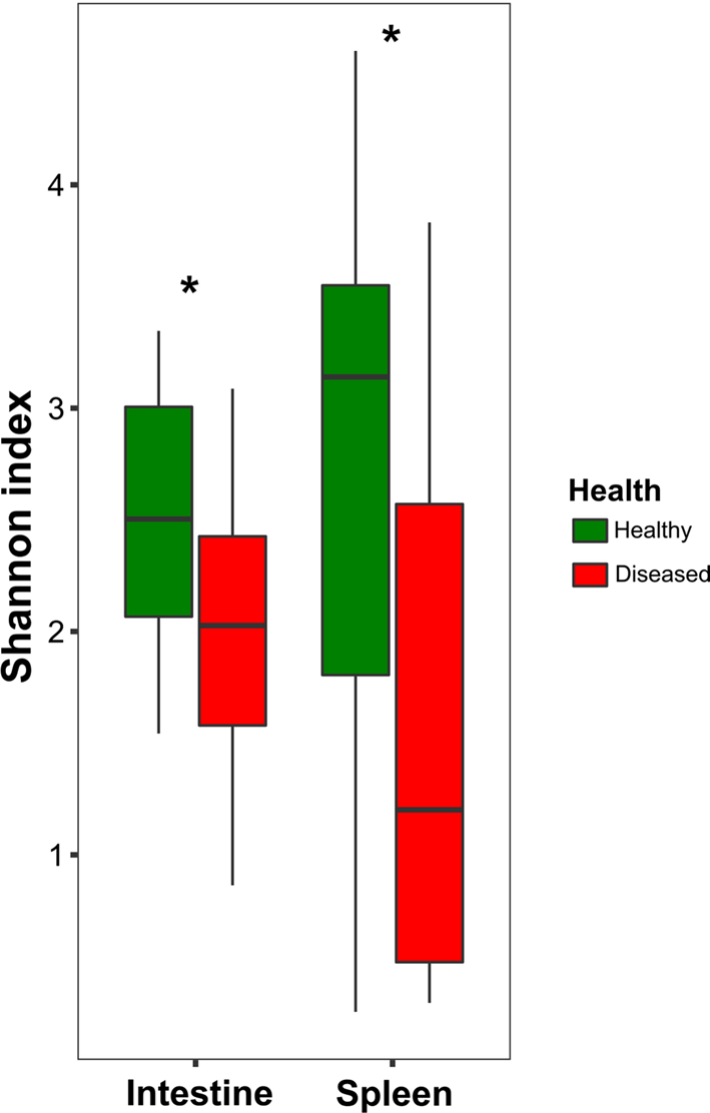
α-diversity (Shannon index) of the microbiota composition (at the ASV level) for intestine and spleen samples from healthy and diseased fish. The diversity was significantly higher in healthy fish compared to the diseased fish in both the intestine and the spleen (ART ANOVA test, F = 8.86, *df* = 1, *p* = 0.005). Asterisks denote significant differences

To examine the effect of fish health on the bacterial diversity in the fish intestine and spleen, the alpha diversity (Shannon index) of the microbiota composition was calculated (Fig. 2). Significantly higher alpha diversity was detected in healthy fish compared to the diseased fish in both the intestine and spleen (F = 8.86, *df* = 1, *p* = 0.005). No significant differences in alpha diversity were found between the two organs of fish with the same health condition (F = 0.05, *df* = 1, *p* = 0.82), and the interaction between the factors (health × organ) was not significant either (F = 2.63, *df* = 1, *p* = 0.11) (Fig. 2).

### Microbiota community composition

**Fig. 3:**
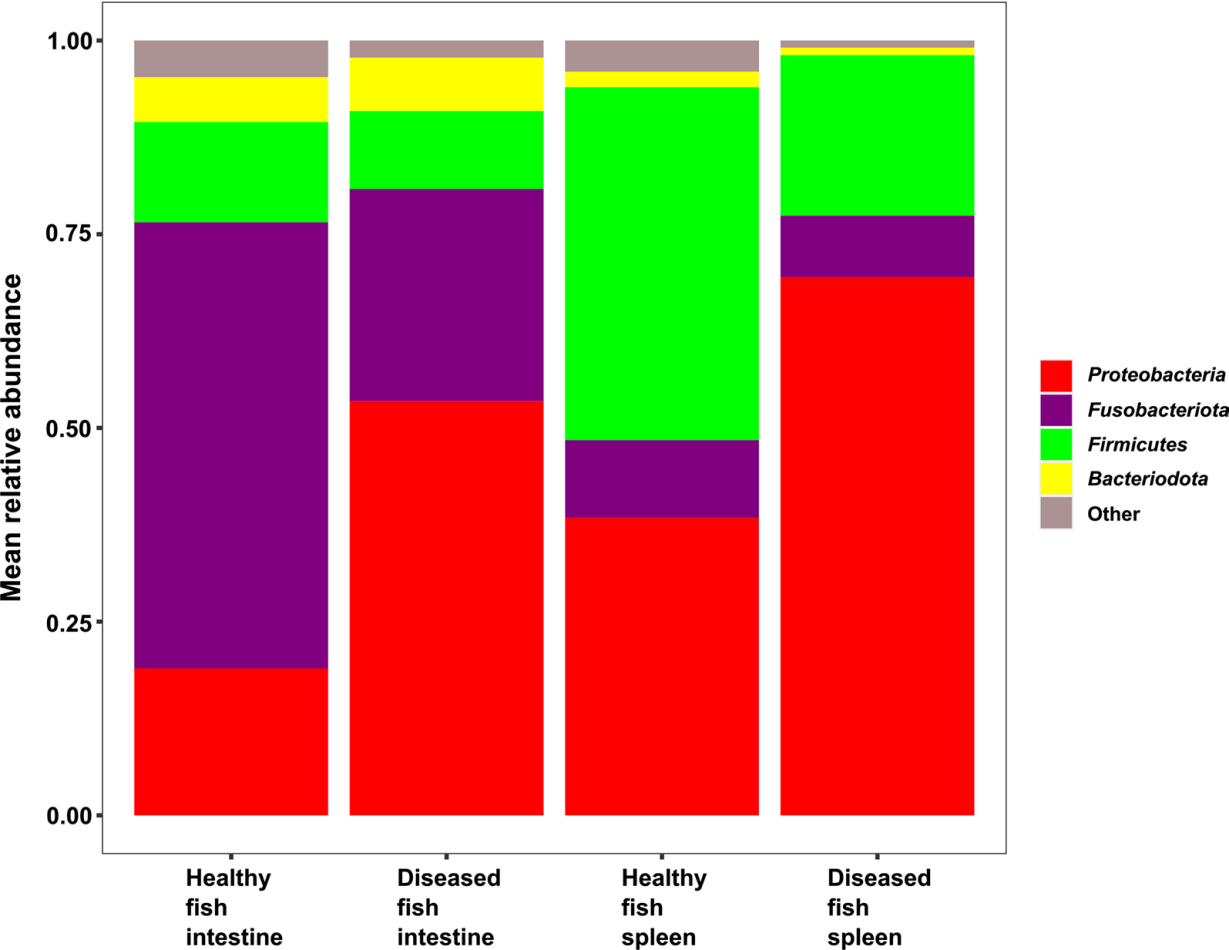
Phylum level mean relative abundance in the intestine and spleen microbiota of healthy and diseased fish

Four dominant phyla (*Proteobacteria, Fusobacteriota, Firmicutes,* and *Bacteroidota*) assembled the microbiota composition of the fish, together reaching a relative abundance from 95.3% in healthy fish intestine samples and up to 99.1% in the diseased fish spleen samples (Fig. 3). In the healthy fish intestine, *Fusobacteriota* was the most dominant phylum with nearly 58.0% mean relative abundance, while the second most dominant phylum was *Proteobacteria* with nearly 19.0% relative abundance. The opposite phenomenon was observed in the diseased fish intestines where *Proteobacteria* was the most dominant phylum with nearly 53.5% mean relative abundance, while the second most dominant phylum was *Fusobacteriota* with 27.0% relative abundance. In both cases, *Firmicutes* was the third most dominant phylum with nearly 13.0% mean relative abundance in healthy fish intestines, and 10.0% in diseased fish intestines. The fourth most dominant phylum was *Bacteroidota* with nearly 6.0% mean relative abundance in the healthy fish intestines and nearly 7.0% in the diseased fish intestines (Fig. 3).

In the healthy fish spleen, *Firmicutes* was the most dominant phylum with nearly 46.0% mean relative abundance, while the second most dominant phylum was *Proteobacteria* with nearly 38.5%. In contrast, in the diseased fish spleen, *Proteobacteria* was the most dominant phylum with nearly 69.5% mean relative abundance, while the second most dominant phylum was *Firmicutes* with 21% mean relative abundance. In both cases, *Fusobacteriota* was the third most dominant phylum with 10.0% and 8.0% mean relative abundance in the healthy and diseased fish spleen, respectively (Fig. 3).

At the genus level (Table 2), *Cetobacterium* was the most dominant in the healthy fish intestines with nearly 51.5% mean relative abundance. Its abundance in diseased fish intestines was reduced by half (26.0%) (Table 2). Another genus that was relatively dominant in the healthy fish intestine was *ZOR0006* with an abundance of 9.0%. *ZOR0006* presence in the diseased fish intestines was about one-third compared to its presence in the healthy fish intestine. In contrast, *Vibrio* was the most dominant genus in diseased fish spleens and intestines with nearly 42.0% and 37.0% mean relative abundance, respectively. *Streptococcus* also characterized diseased fish spleens with about 5.0% abundance (Table 2). More information about the diseased fish samples changes in alpha diversity and the relative abundance of *Vibrio* is provided in (Additional file 1: Table S4).

**Table 2:** Mean relative abundance (percentage ± SE) of the most abundant genera (with at least 5% mean relative abundance in at least one of the fish groups) of the intestinal and spleen microbiota in the studied fish

Order	Family	Genus	Healthy fish intestine	Diseased fish intestine	Healthy fish spleen	Diseased fish spleen
*Fusobacteriales*	*Fusobacteriaceae*	*Cetobacterium*	**51.41 ± 4.87**	**26.09 ± 4.73**	**16.91 ± 4.72**	**10.45 ± 3.05**
*Vibrionales*	*Vibrionaceae*	*Vibrio*	2.21** ± **1.50	**37.35 ± 6.11**	**9.53 ± 7.57**	**41.86 ± 9.25**
*Mycoplasmatales*	*Mycoplasmataceae*	*Mycoplasma*	0.002 ± 0.002	3.69** ± **3.74	**17.17 ± 7.57**	**9.84 ± 4.32**
*Erysipelotrichales*	*Erysipelotrichaceae*	*ZOR0006*	**9.054 ± 3.50**	2.96 ± 1.28	3.93 ± 2.10	1.83 ± 1.15
*Aeromonadales*	*Aeromonadaceae*	*Aeromonas*	2.17 ± 1.21	**6.23 ± 3.51**	**7.10 ± 2.51**	**5.57 ± 3.09**
*Bacteroidales*	*Barnesiellaceae*	Uncl.* (ASV11)	4.63 ± 1.62	**5.49 ± 1.82**	0.72 ± 0.40	1.02 ± 0.74
*Lactobacillales*	*Streptococcaceae*	*Streptococcus*	0.001 ± 0.001	0.62 ± 0.65	0.16 ± 0.09	**5.06 ± 4.55**

Bold values represent mean relative abundance > 5% (For more detailed results see Additional file 2: Data S1)

^*^Unclassified genera

### ASVs that contributed to the variation between organs or health conditions

**Fig. 4:**
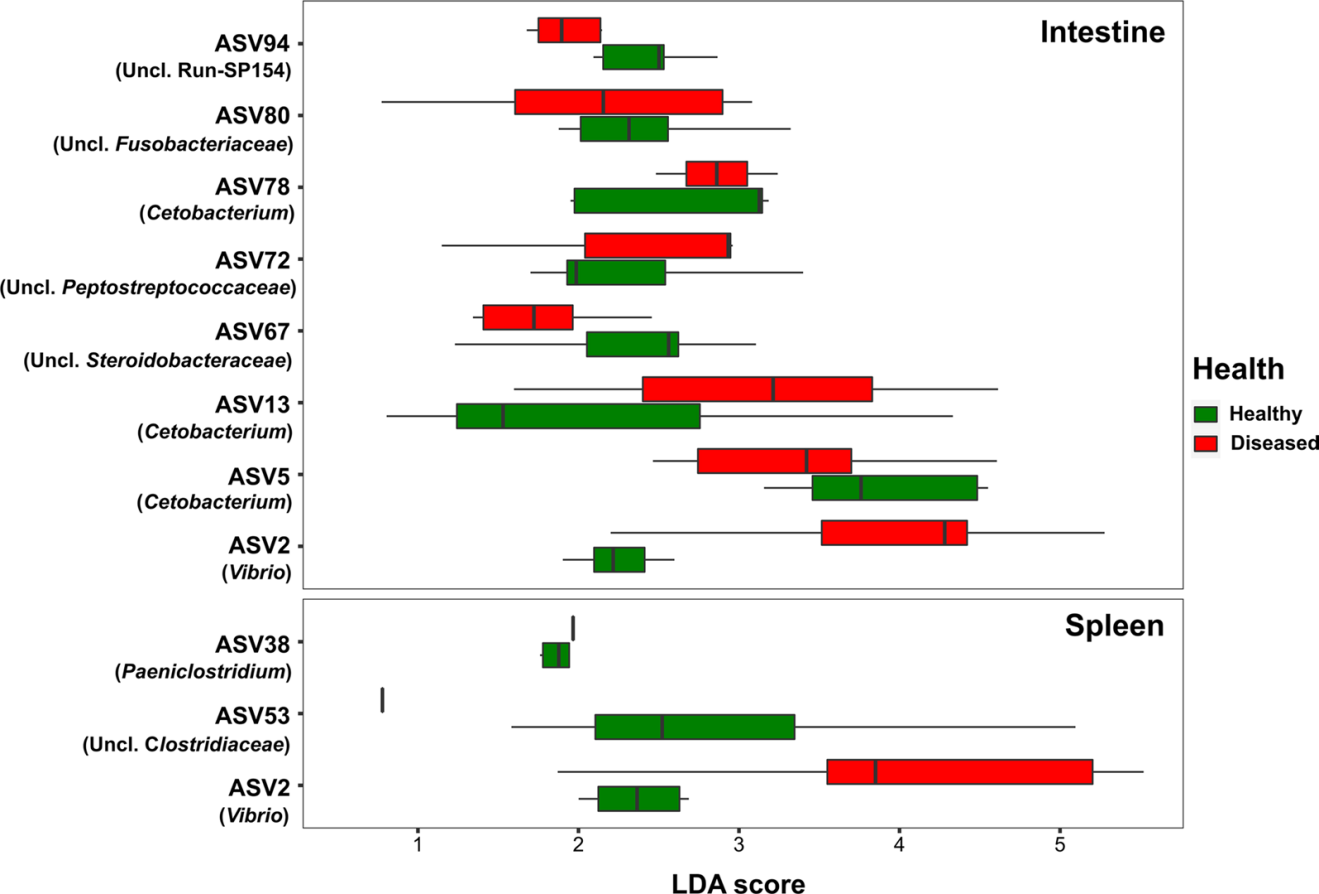
LDA scores of the ASVs from intestines (top) and spleens (bottom) of healthy and diseased fish. The ASVs presented are those with the highest LDA score by LEfSe analysis. Uncl., unclassified

We used the LDA effect size (LEfSe) to identify which ASVs contributed significantly to the variation in the microbiota composition of the intestines and spleens of healthy and diseased fish (Fig. 4).

Eight ASVs contributed significantly to the variation of the microbiota between the healthy and the diseased fish intestines (Fig. 4) while only three ASVs contributed significantly to the variation of the microbiota between the healthy and the diseased fish spleens. In the intestine samples, *Cetobacterium* was represented by three ASVs (ASV5, ASV13, and ASV78) which had relatively high LDA scores in both the healthy and the diseased samples.*Vibrio* (ASV2) was the only ASV that was significant in both the intestine and the spleen samples, with the highest LDA scores in the diseased fish samples.

## Discussion

We studied the intestine and spleen microbiota composition of healthy and diseased hybrid tilapia that were collected from intensive freshwater aquaculture. The fish that showed external signs of disease suffered from parasites and it is possible that the parasites were the primary pathogen that allowed the pathogenic bacteria to invade (Additional file 1: Table S1). Water quality parameters were not measured, and the absence of this data may be a potential limitation of the study.

### Alpha and beta diversity

We demonstrated significant differences in the microbiota composition between healthy and diseased fish in both the intestine and the spleen (Fig. 1 and Table 1). Two recent studies, by Li et al. [22], and Bozzi et al. [19], which compared the intestinal microbiota composition of healthy and diseased largemouth bronze gudgeon (*Coreius guichenoti*) and Atlantic salmon (*Salmo salar*), respectively, also found significant differences between healthy and the diseased intestines. On the other hand, in a study of Ma et al. [20], the differences between the intestinal microbiota composition of healthy and diseased yunlong grouper (*Epinephelus moara*♀ × *Epinephelus lanceolatus*♂), were not significantly different. We also found significant differences in the microbiota between the intestine and the spleen in both healthy and diseased fish (Fig. 1, Table 1.). This is not surprising as the intestine and spleen are organs with very different functions. The fish intestine is a part of the gastrointestinal tract and plays a role in digesting food [32], while the spleen is a blood filter organ that plays a role in the immune system [33].

The alpha diversity (Shannon index) of healthy fish microbiota was higher compared to the microbiota diversity of the diseased fish in both organs (Fig. 2). Moreover, the beta diversity of the intestine and the spleen microbiota was significantly different between the healthy and the diseased fish. Li et al. [22], who studied the intestinal microbiota of healthy and diseased largemouth bronze gudgeon, also found that the microbiota diversity of healthy fish was significantly higher compared to that of diseased fish. On the other hand, Ma et al. [20] did not find significant differences in the diversity of healthy and diseased yunlong grouper. In a review study on the gut microbiota of healthy and diseased fish, Xiong et al. [3] explained that in diseased fish, the invading pathogens compete with the commensal bacteria and reduce the fish gut microbiota diversity.

### The microbiota composition at the phylum level

In the intestine of healthy fish, *Fusobacteriota* was the most dominant phylum, *Proteobacteria* was the second most dominant and *Firmicutes* was the third (Fig. 3). Ofek et al. [6] studied the intestine microbiota composition of hybrid tilapia (among other fish species), and found the same microbiota composition. In the current study (Fig. 3), *Proteobacteria* was more abundant in the intestines of diseased fish (53.4%) compared to their composition in the healthy fish (18.9%). Li et al. [22] also reported that *Proteobacteria* was dominant in the intestinal microbiota of diseased fish (86.0%) compared to healthy fish (55.0%). In the intestine of diseased fish, it appears that the increase in *Proteobacteria* was at the expense of *Fusobacteriota* (Fig. 3).

### The microbiota composition at the genus level

The intestinal microbiota composition of healthy hybrid tilapia was largely comprised by two dominant genera, *Cetobacterium* (51.4%) and *ZOR0006* (9.0%) (Table 2). Similar results were obtained by Ofek et al. [6] for the intestinal microbiota of healthy hybrid tilapia: *Cetobacterium* and *ZOR0006* comprised 60.9% and 11.0% of the microbiota composition, respectively. In their study, Ofek et al. [6] found that *Cetobacterium* was dominant in the intestine of a variety of freshwater fish species. They suggested that *Cetobacterium* might play a role in some intestinal biochemical processes in fish. The results of the current study suggest that *Cetobacterium* plays a role in maintaining a healthy microbiota consortium in the tilapia intestine.

*Vibrio* was the most dominant genus in the diseased fish intestine and spleen (Table 2). This finding implies that a high relative abundance of *Vibrio* in either the fish intestine or spleen, may hint that the fish is sick. Ofek et al. [6], also found that the abundance of *Vibrio* in the intestine of healthy hybrid tilapia was relatively low (0.3%). According to Ina-Salwani et al. [34], vibriosis is one of the most prevalent bacterial fish diseases in aquaculture which affects a variety of fish species. Previous studies reported infection of cultured tilapia by some *Vibrio* species [35–37]. Moreover, Al-Harbi and Uddin [38, 39] isolated *Vibrio* species from diseased hybrid tilapia intestines.

*Mycoplasma* was the most dominant genus in the spleens of healthy fish, the third most dominant in the spleens of diseased fish, much less abundant in the intestine of diseased fish and nearly negligible in the intestine of healthy fish (Table 2). According to Legrand et al. [40], *Mycoplasma* was identified in the intestines of a variety of fish species but not always with association to disease. Bozzy et al. [19], who studied the intestinal microbiota of healthy and diseased Atlantic salmon, reported that *Mycoplasma* was more dominant in healthy fish compared to the diseased fish. Moreover, they found a positive correlation between the abundance of *Mycoplasma* and fish weight, suggesting that *Mycoplasma* was beneficial for the host [19]. El-Jakee et al. [41] isolated *Mycoplasma* from the intestine of moribund tilapia (among other fish species). In the current study, the role of *Mycoplasma* as a pathogenic bacterium remains uncertain.

*Aeromonas* was the third most abundant genus in the intestines of the diseased fish, the fourth in the spleens of healthy and diseased fish and less abundant in the intestines of healthy fish (Table 2). The genus *Aeromonas* is usually part of the fish gut microbiota and has been recognized as a significant pathogen species in aquaculture. *Aeromonas* is generally considered as an opportunistic pathogen, although in some cases it is thought to be a primary pathogens [42, 43]. Recent studies reported disease outbreaks or mortalities of cultured tilapia that were caused by pathogenic *Aeromonas* species [12, 43–46].

*Streptococcus* was detected mainly in the spleens of diseased fish with nearly 5.0% mean relative abundance (Table 2). Streptococcosis is one of the most severe bacterial infections in aquacultures worldwide and causes enormous economic losses [47, 48].

In the current study, most of the diseased fish showed skin hemorrhagic septicemia together with skin lesions and skin necrosis, which are external signs that fish may be infected with *Vibrio* or *Aeromonas* bacteria*.* Only a few of the diseased fish showed eye exophthalmos, which is usually an external sign of Streptococcosis. This is in alignment with the microbiota composition of the diseased fish spleens (Table 2) and may suggest three main pathogenic genera:* Vibrio, Aeromonas* and *Streptococcus* that are responsible for the diseased fish in this study (although colonization of pathogenic bacteria does not always imply infection). Moreover, all the spleen samples of the diseased fish with *Aeromonas* reads, contained *Vibrio* reads as well, which may point at a co-infection between *Vibrio* and *Aeromonas* (Additional file 2: Data S1). Kotob et al. [49], who reviewed co-infections in fish, noted that pathogenic bacterial co-infections in fish, usually have a synergistic interaction. Abdel-Latif et al. [50] noted that the involvement of *Aeromonas* (among other bacteria) in tilapia co-infection is common.

The high LDA scores of *Cetobacterium* and *Vibrio* indicate their contribution to the variation in the microbiota of the intestine and the spleen from healthy and diseased fish. It seems that the main reason for this is the *Cetobacterium* decrease in the intestines of diseased fish compared to the healthy ones and the great increase of *Vibrio* in the intestines and spleens of diseased fish. Ma et al. [20] also reported that *Cetobacterium* decreased from a mean relative abundance of 23% in healthy intestines to 0.5% in diseased intestines.

## Conclusions

There is a significant difference between the microbiota composition of the intestines and spleens of healthy and diseased fish. Significant differences also exist between the two organs of the fish with the same health condition. Diseased fish were characterized by relatively high abundances of *Vibrio*, *Aeromonas* and, *Streptococcus* and thus, their high abundances may indicate illness in hybrid tilapia. Moreover, all the spleen samples of the diseased fish with *Aeromonas* contained *Vibrio* as well, suggesting that they may cause co-infection in fish. *Cetobacterium* and *Vibrio*, which contributed most to the variation in the microbiota of the intestine and the spleen from healthy and diseased fish, respectively, may be used to develop molecular tools for distinguishing between healthy and diseased hybrid tilapia in freshwater aquaculture.

## Supplementary Information

**Additional file 1.** Supplementary Tables and Figures. **Table S1.** Description of fish general health conditions. **Table S2.** Sampling and weight details of the healthy and diseased hybrid tilapia that were included in the current study. **Table S3.** Sample index fish ID, organ, and health condition. **Table S4.** Diseased fish samples that showed the most and least relative abundance of *Vibrio* (total *Vibrio* reads/total reads) and alpha diversity values. **Figure S1.** Maps describing the sampling area. **Fig. S2.** Rarefaction curves representing the observed number of amplicon sequence variants (ASVs) per sample.

**Additional file 2.** **Data S1.** ASV taxonomic classifications and abundances within each fish sample.

## Data Availability

Raw sequence data were submitted to the National Center for Biotechnology Information Sequence Read Archive (https://www.ncbi.nlm.nih.gov/bioproject/) under the BioProject accession number PRJNA753117. Metadata data (read numbers) are given in Additional file 2.
