# Supplementary material for: Intestine and spleen microbiota composition in healthy and diseased tilapia
Source: Anim Microbiome. 2022 Aug 13;4:50. doi: 10.1186/s42523-022-00201-z (PMC9375283; doi:10.1186/s42523-022-00201-z)
Supplement: Supplementary file 1 — Additional file 1. Supplementary Tables and Figures. Table S1. Description of fish general health conditions. Table S2. Sampling and weight details of the healthy and diseased hybrid tilapia that were included in the current study. Table S3. Sample index fish ID, organ, and health condition. Table S4. Diseased fish samples that showed the most and least relative abundance of Vibrio (total Vibrio reads/total reads) and alpha diversity values. Figure S1. Maps describing the sampling area. Fig. S2. Rarefaction curves representing the observed number of amplicon sequence variants (ASVs) per sample. [file 42523_2022_201_MOESM1_ESM.docx]

**Additional file 1**

Supplementary Tables and Figures

**Table S1.** Description of fish general health conditions. More details regarding the fish identity (ID) can be found in Table S3 and in Additional file 2: Data S1.

| **Fish IDs** | **Health**  **status** | **External signs of disease** | **Parasites** | **Parasites**  **level** | **Bacteriological**  **results** |
| --- | --- | --- | --- | --- | --- |
| Ti 1,2,6,11,14, 15,16 | Diseased | Skin lesions, skin necrosis and hemorrhagic septicemia | *Trichodina* sp*.* | **+++** | Motile rods |
| Ti 20,21,22 | Diseased | Skin lesions, skin necrosis and hemorrhagic septicemia | *Trichodina* sp*.*  *Gyrodactylus* sp.  *Sessilina* sp*.* | **+++++**  **+++**  **+++** | Motile rods |
| Ti 26,27,28,31, 32,34,35,36,37 | Diseased | Skin lesions, skin necrosis and hemorrhagic septicemia | *Trichodina* sp*.*  *Sessilina* sp*.* | **++++**  **++++** | Motile rods |
| Ti 3,33, 34 | Diseased | Eye exophthalmos | *Trichodina* sp*.* | **+++** | *Streptococcus* sp*.* |
| Ti 7,12,13,17, 18,19,23,24, 25,29,30,39 | Healthy | No signs | *Trichodina* sp*.*  *Sessilina* sp*.* | **+**  **+** | **-** |
| Ti 4,5 | Healthy | No signs | No parasites | **-** | **-** |

**Table S2.** Sampling season and weight details of the healthy and diseased hybrid tilapia sampled in the current study.

| **Health** | **n** | **Sampling season**  **(number of fish)** | **Average weight**  **(gr) (± SD)** |
| --- | --- | --- | --- |
| Healthy | 14 | Winter (n=4), Spring (n=5), Summer (n=5) | 490.78 **±** 174.76 |
| Diseased | 22 | Winter (n=7), Spring (n=4), Summer (n=11) | 624.90 **±** 205.52 |

**Table S3.** Samples index, fish ID, organ, and health condition.

| **Fish ID** | **Health** | **Sample** | **Organ** | **Sample** | **Organ** |
| --- | --- | --- | --- | --- | --- |
| Ti1 | Diseased | A3 | Intestine | A4 | Spleen |
| Ti2 | Diseased | A10 | Intestine | A7 | Spleen |
| Ti3 | Diseased | A16 | Intestine | A13 | Spleen |
| Ti4 | Healthy | B5 | Intestine | B2 | Spleen |
| Ti5 | Healthy | B10 | Intestine | B8 | Spleen |
| Ti6 | Diseased | C4 | Intestine | C2 | Spleen |
| Ti7 | Healthy | - | - | C7 | Spleen |
| Ti11 | Diseased | E4 | Intestine | E1 | Spleen |
| Ti12 | Healthy | E10 | Intestine | E7 | Spleen |
| Ti13 | Healthy | E16 | Intestine | E15 | Spleen |
| Ti14 | Diseased | F4 | Intestine | F2 | Spleen |
| Ti15 | Diseased | F10 | Intestine | F7 | Spleen |
| Ti16 | Diseased | F16 | Intestine | F13 | Spleen |
| Ti17 | Healthy | F23 | Intestine | F19 | Spleen |
| Ti18 | Healthy | F28 | Intestine | F25 | Spleen |
| Ti19 | Healthy | F34 | Intestine | F31 | Spleen |
| Ti20 | Diseased | G1 | Intestine | G4 | Spleen |
| Ti21 | Diseased | G10 | Intestine | G7 | Spleen |
| Ti22 | Diseased | G16 | Intestine | G13 | Spleen |
| Ti23 | Healthy | G22 | Intestine | G20 | Spleen |
| Ti24 | Healthy | G28 | Intestine | G27 | Spleen |
| Ti25 | Healthy | G35 | Intestine | - | - |
| Ti26 | Diseased | H4 | Intestine | H2 | Spleen |
| Ti27 | Diseased | H10 | Intestine | H7 | Spleen |
| Ti28 | Diseased | H16 | Intestine | H13 | Spleen |
| Ti29 | Healthy | H22 | Intestine | H19 | Spleen |
| Ti30 | Healthy | - | - | H25 | Spleen |
| Ti31 | Diseased | H34 | Intestine | H31 | Spleen |
| Ti32 | Diseased | I4 | Intestine | I1 | Spleen |
| Ti33 | Diseased | I10 | Intestine | I7 | Spleen |
| Ti34 | Diseased | J4 | Intestine | J1 | Spleen |
| Ti35 | Diseased | J10 | Intestine | J7 | Spleen |
| Ti36 | Diseased | K4 | Intestine | K1 | Spleen |
| Ti37 | Diseased | K10 | Intestine | K7 | Spleen |
| Ti38 | Diseased | K16 | Intestine | - | - |
| Ti39 | Healthy | - | - | K21 | Spleen |

**Table S4.** Diseased fish samples that showed the most and least relative abundance of *Vibrio* (total *Vibrio* reads/total reads) and alpha diversity values. More details regarding the fish identity (ID) can be found in Table S3 and in Additional file 2: Data S1.

| **Sample ID** | **Organ**  **(Diseased)** | ***Vibrio* relative abundance** | **Alpha diversity** |
| --- | --- | --- | --- |
| H13 | Spleen | 99.76% | 0.45 |
| H7 | Spleen | 99.65% | 0.31 |
| H31 | Spleen | 99.35% | 0.05 |
| I1 | Spleen | 98.39% | 0.33 |
| F13 | Spleen | 98.13% | 0.38 |
| H10 | Intestine | 0.64% | 2.23 |
| K7 | Spleen | 0.62% | 1.66 |
| J7 | Spleen | 0.51% | 1.92 |
| C2 | Spleen | 0.00% | 1.24 |


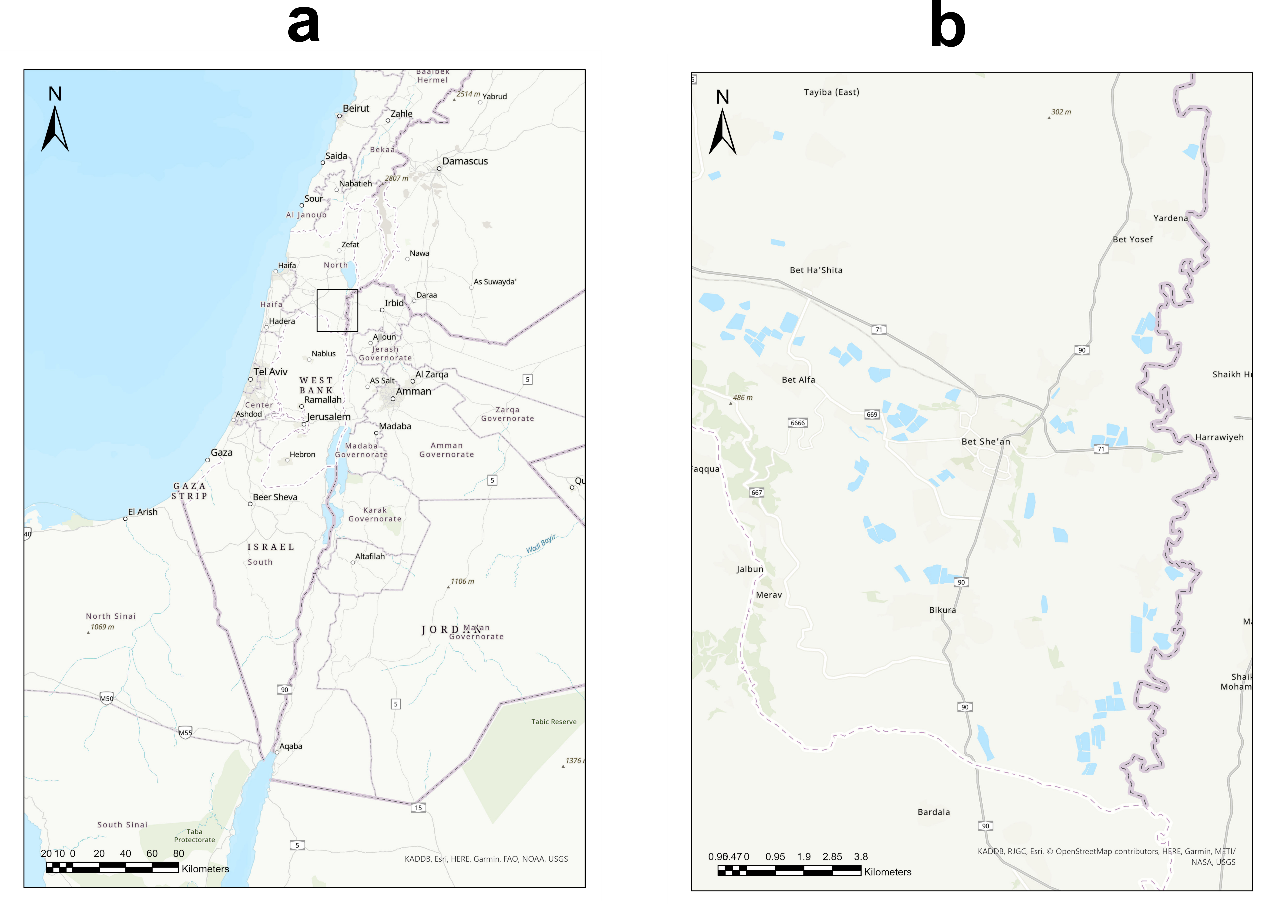


a

b

**Figure S1.** Maps describing the sampling area. (a) A map of Israel. The sampling area is marked in red (b) A zoomed-in map of the fishpond sampling area that is marked in red in map a**.** The map was modified from: KADDB, Esri, HERE, Garmin, FAO, NOAA, USGS and from KADDB, RJGC, Esri, © OpenStreetMap contributors, HERE, Garmin, METI/NASA, USGS.

**
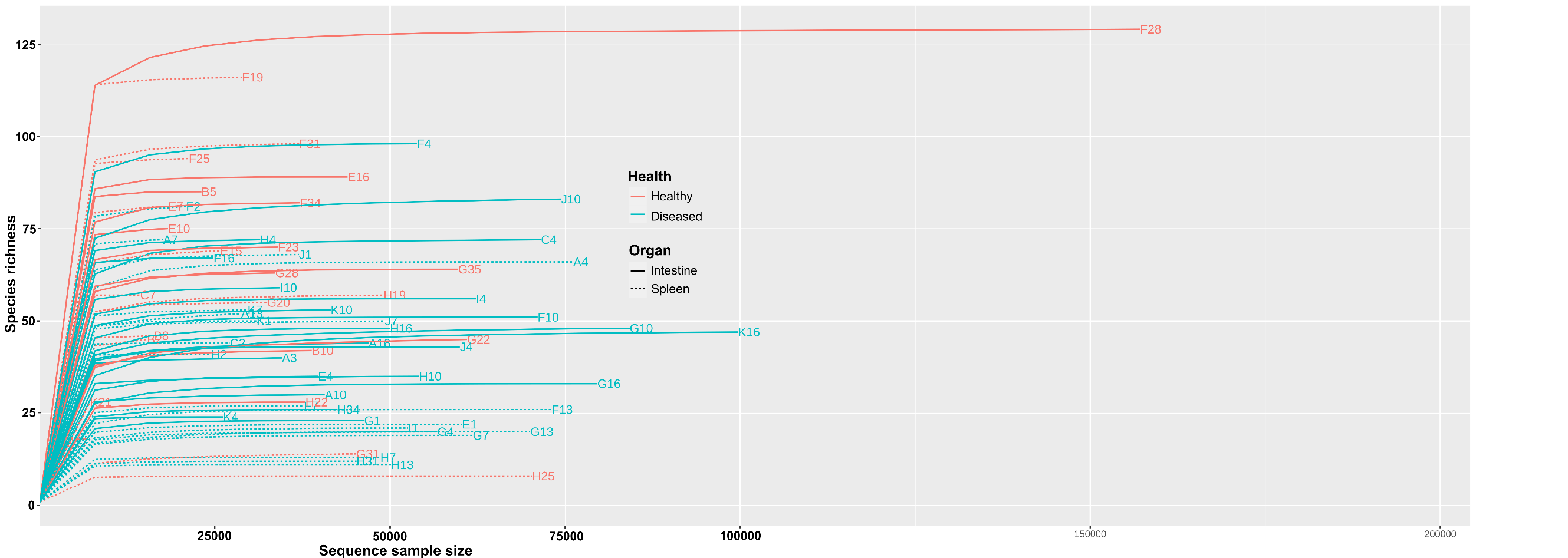
**

**Fig. S2.** Rarefaction curves representing the observed number of amplicon sequence variants (ASVs) per sample. Rarefaction curves present the increase in the number of the ASVs as a function of the sequencing depth for each sample.
